# Contamination-free graphene by chemical vapor deposition in quartz furnaces

**DOI:** 10.1038/s41598-017-09811-z

**Published:** 2017-08-30

**Authors:** Nicola Lisi, Theodoros Dikonimos, Francesco Buonocore, Martina Pittori, Raffaello Mazzaro, Rita Rizzoli, Sergio Marras, Andrea Capasso

**Affiliations:** 1 Energy Technologies Department (DTE), ENEA Casaccia, Via Anguillarese 301, 00123 Rome, Italy; 2 Department of Chemical Materials Environmental Engineering, Sapienza University of Rome, Via del Castro Laurenziano 7, 00161 Rome, Italy; 3 Institute for Microelectronics and Microsystems (IMM), National Research Council (CNR), Section of Bologna, Via Gobetti 101, 40129 Bologna, Italy; 4 Chemistry Department ‘G. Ciamician’, Bologna University, Via Selmi 2, 40126 Bologna, Italy; 5 Istituto Italiano di Tecnologia, Materials Characterization Facility, Via Morego 30, 16163 Genova, Italy; 6 Istituto Italiano di Tecnologia, Graphene Labs, Via Morego 30, 16163 Genova, Italy

## Abstract

Although the growth of graphene by chemical vapor deposition is a production technique that guarantees high crystallinity and superior electronic properties on large areas, it is still a challenge for manufacturers to efficiently scale up the production to the industrial scale. In this context, issues related to the purity and reproducibility of the graphene batches exist and need to be tackled. When graphene is grown in quartz furnaces, in particular, it is common to end up with samples contaminated by heterogeneous particles, which alter the growth mechanism and affect graphene’s properties. In this paper, we fully unveil the source of such contaminations and explain how they create during the growth process. We further propose a modification of the widely used quartz furnace configuration to fully suppress the sample contamination and obtain identical and clean graphene batches on large areas.

## Introduction

Graphene – a single atomic layer of carbon atoms tightly packed in a two-dimensional honeycomb lattice, chemically inert with super strength, and the ability to conduct heat and electricity better than any other known material – has been suggested for a variety of potential industrial applications, ranging from flexible electronics to high-speed computing, and from more efficient wind turbine blades to next-generation solar cells^1–6^. Concerning the applications in electronics, the fabrication of low-cost, large-area, high-quality graphene still poses crucial challenges. Since graphene was first isolated by mechanical exfoliation^1^, different production methods have been explored, which can used for diverse application^7–10^. Among the many, chemical vapor deposition (CVD) is currently considered the optimal method for producing graphene for high-performance electronics and on a large scale, considering its accessible cost, wide scalability and the high crystalline quality achievable^9^. In a typical CVD process, carbon-bearing gases react at high temperatures (900–1100 °C) in the presence of a metal catalyst, which serves both as a catalyst for the decomposition of the carbon species and as a surface for the nucleation and accretion of the graphene lattice^11^. The details of the graphene growth process are articulated and still subject of intense study^12^, being several factors involved: (i) the metal catalyst with its surface morphology (e.g., macro/micro structure, faceting, defectiveness) and properties (e.g., catalytic activity, evolution upon heating, solubility of carbon atoms into the bulk)^13–19^; (ii) the choice of carbon precursor^20^; (iii) the CVD parameters, especially temperature and pressure. Copper (Cu) and nickel (Ni) are at present the preferred metal substrates because of their favorable catalytic properties and low cost. Cu in particular is advantageous as it is easily removed after the growth, hence offering an efficient means for graphene transfer^11^. Methane is the most used carbon precursor^9, 11^, although many other carbon sources have proven very efficient in later years: Among them ethanol is a valid choice for the growth of high-quality graphene at high temperature (>1000 °C)^21, 22^. As a carbon precursor, ethanol is safer and easier to handle than methane, being liquid at STP. Advantages arise also from the weakly oxidizing ability of ethanol, which translates during the CVD process in an increased graphene growth rate (faster than in the case of methane), and hence in the formation of continuous graphene layers in just a few seconds^23^. Ethanol can yield large graphene crystals inside “sealed” Cu enclosures with a quality comparable to those obtained with methane precursor^24^. It also contains oxygen, whose beneficial effect in reducing the nucleation density and in improving the quality of the grown graphene has been recently recognized and explored. A pure oxygen flow prior to the growth or the use of so-called oxygen-rich copper substrates were proved to favor the growth of graphene of higher quality than pure hydrocarbons^25^. The presence of some amounts of carbon dioxide, a milder oxidant, was also found to suppress unwanted graphitic phases^26^.

Usually, CVD graphene is grown on Cu foils at high temperature (typically 1000 °C), slightly below the metal’s melting temperature (T_m_ = 1084.62 °C). CVD can be performed either in cold-wall metal chamber reactors or in hot-wall reactors. The latter kind of reactors are the most widespread because of the steady-state, isothermal, clean gas flow conditions they offer, which can be achieved by simple and inexpensive experimental instrumentations. A hot-wall reactor usually consists of a quartz-tube furnace^9, 11, 12^, where by “quartz” the graphene community is commonly intending fused quartz or fused silica, i.e., amorphous SiO_2_. Differently from other kind of glasses, fused quartz contains no heterogeneous elements and then has higher working and melting temperatures^27^, with negligible vapor pressure at 1000 °C^28^. A tube-furnace made of electrically fused quartz of the highest grade can operate in vacuum up to 1160 °C. For these reasons most of the research focusing on the CVD growth of graphene on Cu has been carried out in hot-wall quartz furnaces. Although excellent in terms of crystalline quality, the graphene samples grown with this kind of CVD systems may often show dot-like contaminations on their surface. These contaminations appear as white particles (with various density) sitting on top of graphene in the reported scanning electron microscopy (SEM) images (either on Cu or on other transfer substrates), or are identifiable as round particle with height of a few nm in atomic force microscopy (AFM) images. The contaminations are found when graphene is grown in a CVD system with a quartz tube (either at low or atmospheric pressure), regardless of the carbon precursors^29–36^, although their density seem to be higher in case of oxygen-containing precursors^34, 36^. No definitive explanation for this contamination issue has been so far presented, nor sufficient emphasis has been given, in our opinion, to such relevant and ubiquitous effect.

Only very recently, a few groups analyzed and discussed the nature of such contamination. Ruiz *et al*. identified the contaminants as SiO_2_ clusters and indicated the phase transition of quartz (occurring during the CVD process) as responsible for the contamination process: SiO_2_ is emitted from the quartz tube when Cu atoms (originating from the Cu substrates during heating) diffuse inside the tube walls at the α/β quartz phase transition temperature (573 °C)^37^. Other authors attributed the SiO_x_ contamination to the presence of silicon (Si) in the bulk of the Cu substrates, which in their view would emerge during the growth process and contaminate the graphene surface^38^. A third group linked the presence of SiO_2_ clusters on graphene to the reaction of hot hydrogen with the quartz reactor^39^: During the annealing of the Cu substrate the hot part of the tube furnace would be etched by H_2_ gas, depositing SiO_2_ on the substrate. These studies propose possible contamination mechanisms but leave some questions unanswered. In the first case, it is unclear how the α/β transition may occur in a tube made of amorphous fused quartz. In the second, if the bulk of the Cu catalyst is the source of SiO_x_ particles, it is difficult to explain how these particles can be found on top of graphene if they emerge from the underlying Cu substrate (how SiO_x_ forms from Si during the CVD process should also be explained). Concerning the last study, the proposed mechanism is not supported by any other evidence or published record for the reaction of quartz with molecular hydrogen at 1000 °C.

In this paper we discuss the contamination issue and its effect on the production of graphene, presenting a practical solution to solve it and obtain clean graphene in a consistent and reproducible way. In our case study, we focus on graphene grown on copper foils using ethanol as liquid precursor^21^, which, as mentioned, has been observed to lead to a high level of contamination^35^. The presence of contaminants in the grown graphene is assessed by electron and scanning probe microscopy, and by energy dispersive X-ray spectroscopy. We ascertain the contamination mechanism and ultimately propose a novel design for a CVD system capable of completely eliminating the occurrence of contamination.

## Results and Discussion

### SiO_2_ contaminated graphene

**Figure 1:**
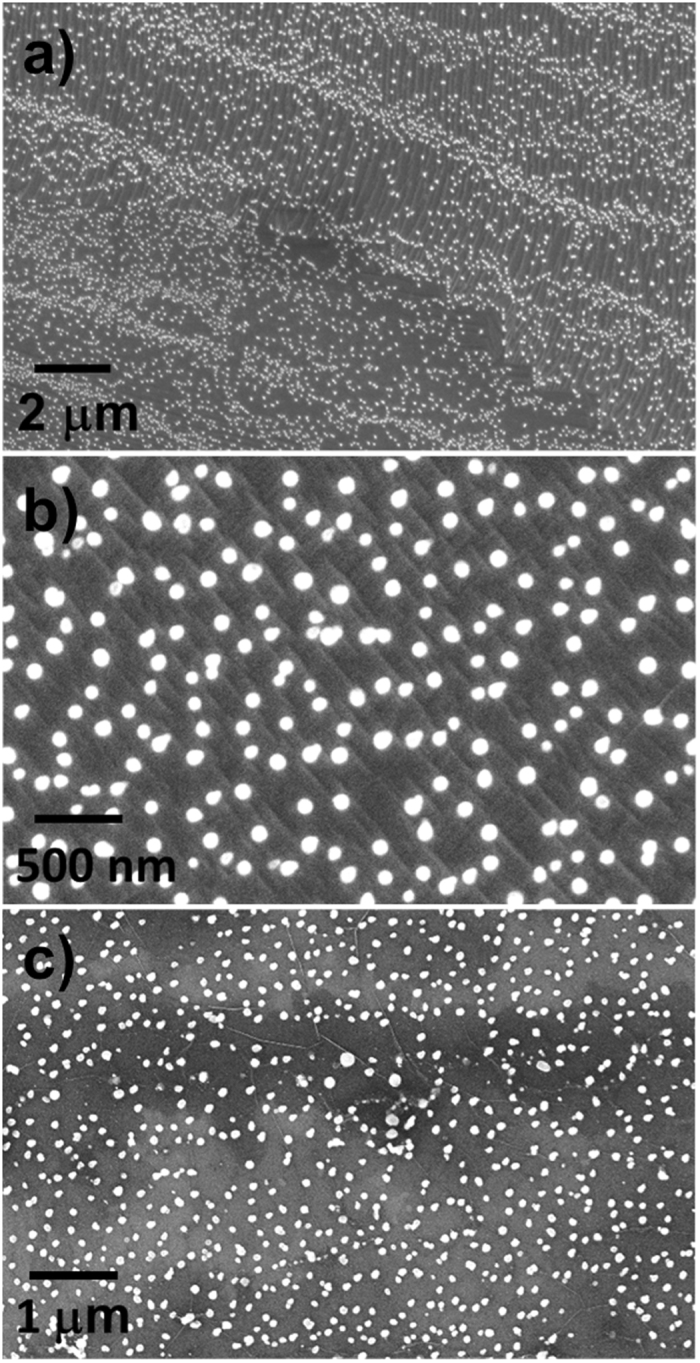
SEM images of a heavily contaminated graphene film grown by CVD with an aged quartz tube, on copper (**a**,**b**) and after transfer on Si/SiO_2_ 300 nm (**c**). Cu surface reconstruction and graphene folds are clearly visible.

This study began after the observation of microscopic particles dispersed on the graphene samples grown with our CVD system, which has the typical configuration (see Experimental) detained by most of the CVD systems used in literature for graphene growth^30–39^. At the time of that first observation, our quartz furnace had previously sustained hundreds of graphene growth processes (and hence a corresponding number of heating/cooling cycles). Heavy contaminations (a particle density up to 10^14^ cm^−2^) could be found in all the graphene samples, with the highest density in case of the longest growth processes (30 min). Figure 1a,b show SEM micrographs of graphene samples on copper substrates after the growth: The contaminations appear as small, rounded white dots over the surface. They are preserved after transferring the graphene films onto Si/SiO_2_, as visible in Fig. 1c. Such a substantial degree of contamination was found to be connected to the working age of the quartz tube. A proof of this is the fact that no contaminations could be found on graphene when installing a new tube (along with a new quartz boat) in the CVD system (Figure S1); with a new tube, uncontaminated graphene samples could be produced until a given number of growth processes were done. The microscopic analysis of the graphene samples transferred on Si/SiO_2_ showed a degree of contamination increasing with the working age of the tube. To further evaluate the impact of the quartz tube ageing on the sample pollution, we examined graphene grown in quartz tubes with an increasing number of operating hours, evaluated in terms of heating/cooling cycles. In general, when using quartz tubes with a few tens of cycles graphene appears clean of contamination upon SEM and AFM inspection. The contamination increases from negligible to noticeable, finally becoming very heavy; three broad ranges have been identified: <50 cycles, ~100 cycles, and >150 cycles. The contamination level was observed to worsen in case of longer (>30 min) and lower pressure (<1 mbar) process, not reported here.

**Figure 2:**
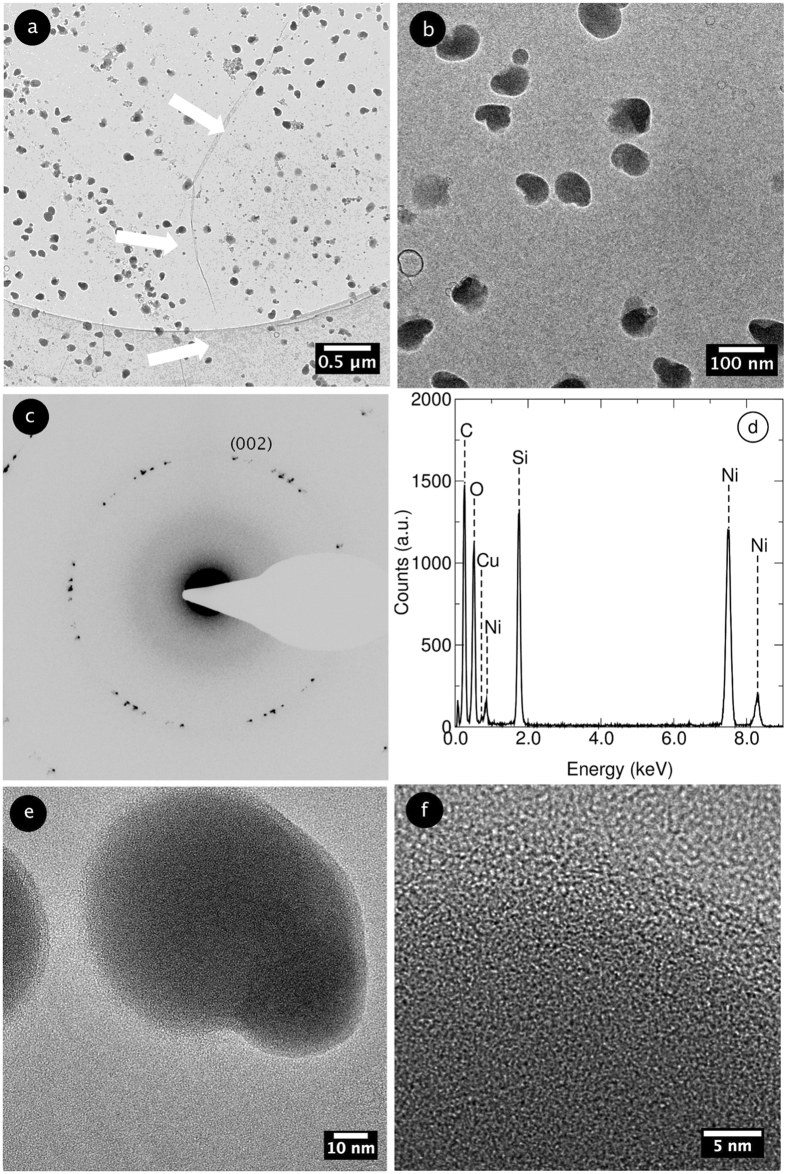
(**a**,**b**) TEM images of contaminated graphene with different magnifications. The white arrows indicate the typical wrinkles of CVD graphene. (**c**) Diffraction pattern taken from the image in (**b**). (**d**) EDX spectrum taken in the contaminated region (**b**). (**e**,**f**) HRTEM images of the contaminants at different magnifications.

Transmission electron microscopy (TEM) was employed to analyze the impurities. Figure 2a and b show TEM bright field micrographs of a contaminated graphene film at different magnifications. The contaminants are visible as dark, bean-shaped particles, ubiquitous all over the graphene film. The diffractogram in Fig. 2c reports the different sets of spots corresponding to the (002) graphite fringes on the typical hexagonal pattern, thus clearly showing the graphene’s poly-crystalline structure. From the Energy dispersive X-ray spectroscopy (EDX) pattern in Fig. 2d it is evident the presence of silicon and oxygen associated to the contaminants (the Ni peaks originate from the TEM grid). The HRTEM images in Fig. 2e and f show the lack of a dominant crystalline phase of the SiO_x_ particles, which can be thus deemed to be amorphous.

### Contamination mechanism

**Figure 3:**
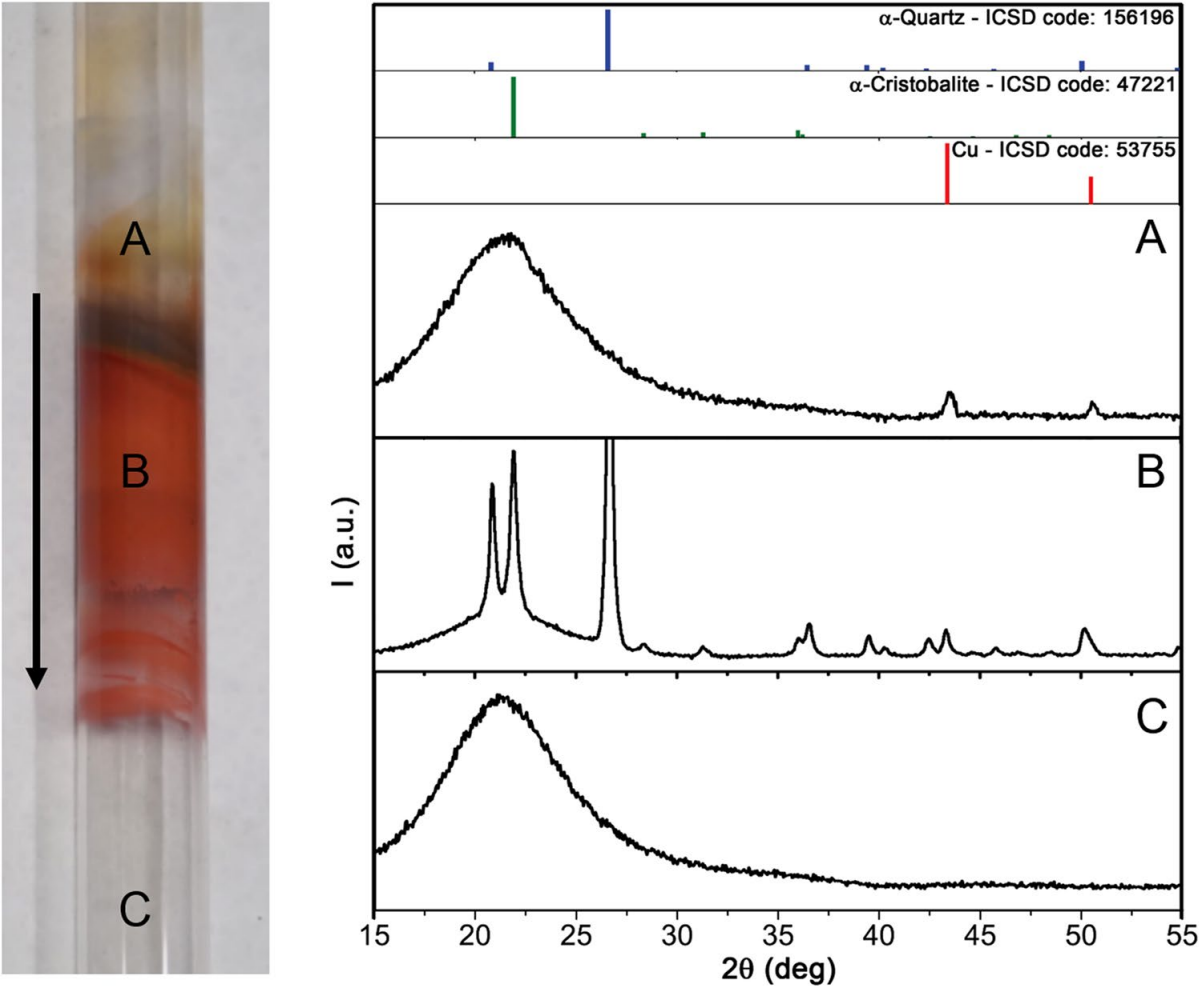
XRD patterns taken in parts of the aged quartz tube (in the photograph, with the arrow indicating the gas flow direction) reaching different temperatures during the graphene growth process: (**A**) 570 °C, (**B**) 860 °C, (**C**) 1070 °C. Before the analysis the inner tube surface has been cleaned with nitric acid to remove the condensed Cu. The colors of the different tube regions (red for metallic Cu or cuprous oxide - Cu_2_O -, and black for cupric oxide - CuO) demonstrate the diffusion into the bulk quartz along a temperature gradient.

Quartz has a framework structure of SiO_4_ tetrahedra linked by shared ‘corner’ oxygen atoms. The crystalline polymorphs of quartz undergo a reversible phase transition at 573 °C, where the lattice structure changes from α- to β-quartz (a rearrangement from trigonal to hexagonal phase called “inversion”)^40, 41^. Concurrently, the quartz lattice expands by a factor of 0.45%, with a density decrease from 2.65 g/cm^3^ to 2.53 g/cm^3^. As mentioned in the introduction, it has been suggested that these density fluctuations are responsible for the diffusion of Cu into the bulk of the quartz tube and the consequent displacement of SiO_2_, which is emitted and eventually deposits on graphene as nanoparticles^37^. This explanation is however incomplete, since pure amorphous fused silica does not undergo phase transitions within the usual CVD temperature range (up to ~1070 °C). During CVD, however, the quartz tube gets in contact with impurities and reactive elements and is subject to heating/cooling phases at varying pressure conditions: Therefore a more complex scenario involving “devitrification” should be taken into consideration. Devitrification is a recrystallization process occurring above ~1220 °C^27^ in fused silica via the nucleation and growth of metastable crystalline phases (i.e., cristobalite). Although even the highest graphene growth temperatures on Cu substrates are not sufficient to enable the devitrification of pure fused silica, the presence of impurities (such as Cu atoms) can trigger it even at temperatures around 600 °C^42^. The amorphous structure of fused silica could be further degraded by repeated exposure to oxidizing and reducing environments^43^, a conditions that is met during CVD and that may also contribute to a faster devitrification kinetics^44^. In order to ascertain and understand the possible structural evolution of the CVD quartz tube, we analyzed it by X-ray diffraction (XRD) after >150 graphene CVD growth processes. We concentrated the analysis on three tube parts held at different temperatures in the furnace during CVD, as detailed in Fig. 3.

The XRD pattern of the tube area held at the graphene growth temperature (part C) shows the broad halo typical of amorphous quartz. This pattern is coincident to that of the room-temperature areas of the tube (not shown). The tube part A shows an analogous amorphous quartz pattern but in this case two weak peaks ascribed to metallic Cu also appear. Conversely, the tube part B presents a completely different spectrum: two set of peaks ascribed to α-quartz and α-cristobalite are accompanied by a small amount of metallic Cu. This analysis sheds light on the evolution of the quartz phases due to the CVD processes. Although the hot tube zone reaches temperatures up to 1070 °C, no Cu atoms (sublimating from the foil substrate) condense on its inner surface and thus neither Cu diffusion nor devitrification take place. On the tube zone held up to 570 °C, Cu can condense and diffuse into the bulk, but the temperature is not high enough for the devitrification to occur. The situation is completely different for the tube zone kept at temperature up to 860 °C, where the Cu atoms are able to diffuse and promote the devitrification^42^. The α-cristobalite phase formed by the devitrification is metastable and slowly transforms in β-quartz, and then back to α-quartz upon cooling to room temperature. We further confirmed such phase transformations by checking the evolution of the XRD spectrum of an analogous quartz tube part while heating in vacuum up to 900 °C, as shown in Figure S2. These XRD analyses allow to explain the SiO_x_ contamination of CVD graphene, as follows.

The diffusion of Cu atoms into the quartz tube (which in principle can occur regardless of the quartz crystallinity^45, 46^) is fostered during CVD by the α/β quartz phase transitions induced by the devitrification. While diffusing within the bulk, the Cu atoms subtract oxygen from the quartz (silica) forming SiO, which is emitted as vapor into the tube. This happens since the SiO vapor pressure is two orders of magnitude or more higher than that of SiO_2_^27, 47^. The gaseous “desiliconization” is an established technique for the removal of silica from minerals and ores^48^. The emitted SiO vapor eventually condense and deposit as nanoparticles inside the CVD chamber, polluting the Cu substrates and the growing graphene. Such contamination issue is so serious and ubiquitous that we deem it partially responsible for the common choice in literature of the semi-sealed “copper enclosures” used as growth substrates in fundamental studies about the nucleation and growth of graphene^24, 25^. Curiously, this contamination process has been even exploited as a source for the controlled assembly of SiO_x_ nanoparticles, aiming at the formation of lateral heterostructures between SiO_x_ and graphene^49^. Other than clearly being an unwanted contamination, these nanoparticles are expected to affect the graphene growth process, e.g., by acting as preferential nucleation sites and/or undesired secondary nucleation sites (giving rise to multilayered graphene in an otherwise self-limiting monolayer growth process)^31^. This situation is further confirmed by inspecting CVD graphene grown in our aged quartz tube (Figure S3), where SiO_x_ particles are systematically found within secondary graphene domains, highlighting their role as preferential/additional nucleation sites. A obvious way of overcoming this situation would imply the frequent substitution of the used quartz glassware, with a high impact of the production cost and flexibility.

### Contaminant-free growth

**Figure 4:**
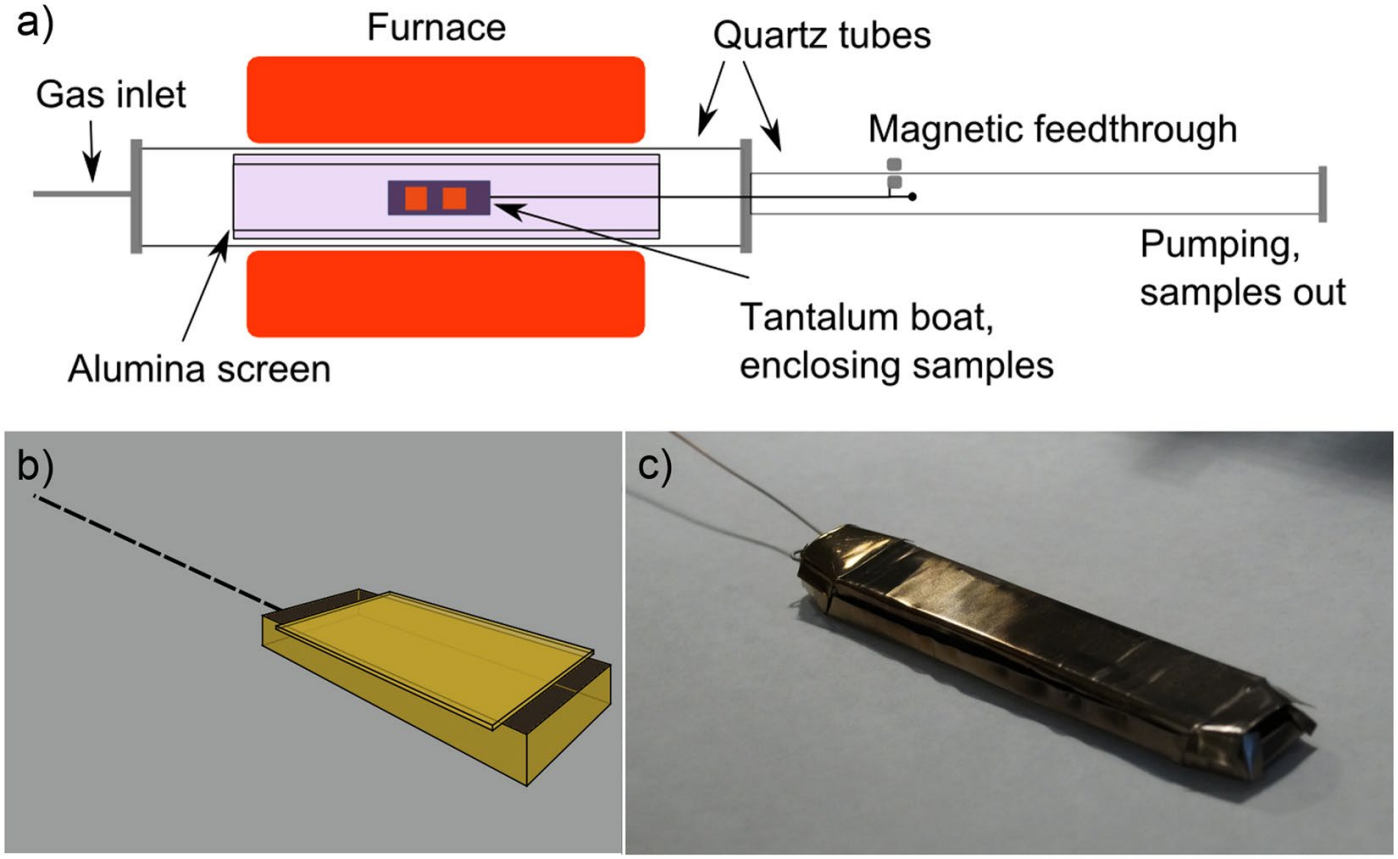
(**a**) Schematics of the modified configuration of the low-pressure CVD system with a coaxial alumina screen tube. The boat is inserted and extracted from the hot zone as in the original configuration (see Experimental). (**b**) Graphic design and (**c**) photographic image of the tantalum boat with lid (12 cm in length).

In order to overcome the practical and cost limitations arising from replacing the quartz tubes every few growth processes, we developed a novel reactor configuration by adding an alumina tube coaxial to the tube furnace as a screen for Cu (from the substrates to the tube) and SiO (from the tube to the substrate) vapors contaminants during growth (schematics in Fig. 4). An alumina “boat” was used instead of the quartz one in order to suppress its contribution to the sample contamination (as discussed in the SI). Alumina ceramic possesses a remarkably higher thermo-chemical stability and its melting temperature^50^ (2070 °C) is considerably higher than that of quartz. Conversely, a tube made of alumina would be less proficient in a CVD furnace configuration due to many practical drawbacks with respect to one made of quartz: Alumina’s higher thermal conductivity would increase the heat flow along the tube axis; its higher sensitivity to thermal shocks could cause mechanical failures and its rough, microcrystalline surface would make vacuum head seals more difficult to realize. We avoided all these issues by using a larger quartz tube (60mm outer diameter - OD - and 55mm inner diameter - ID) fitted with high vacuum seals and a smaller coaxial alumina tube (40mm OD, 30mm ID), bridging the two reactor cold ends and acting as a screen (Fig. 4). In this geometry, the SiO vapors cannot travel directly from the inner quartz tube surface towards the hot samples since they would readily condense onto the colder alumina tube edges, nor Cu vapors could follow the opposite route to coat and degrade the quartz walls. Alumina is not impervious to copper, which at high temperature can diffuse into its bulk^51^, however the diffusion coefficient is considerably lower than that in SiO_2_^45^; further, alumina is not subject to phase transitions at pressures and temperatures typical for CVD. In Figure S4, it is possible to appreciate the reduction of contamination at this stage.

**Figure 5:**
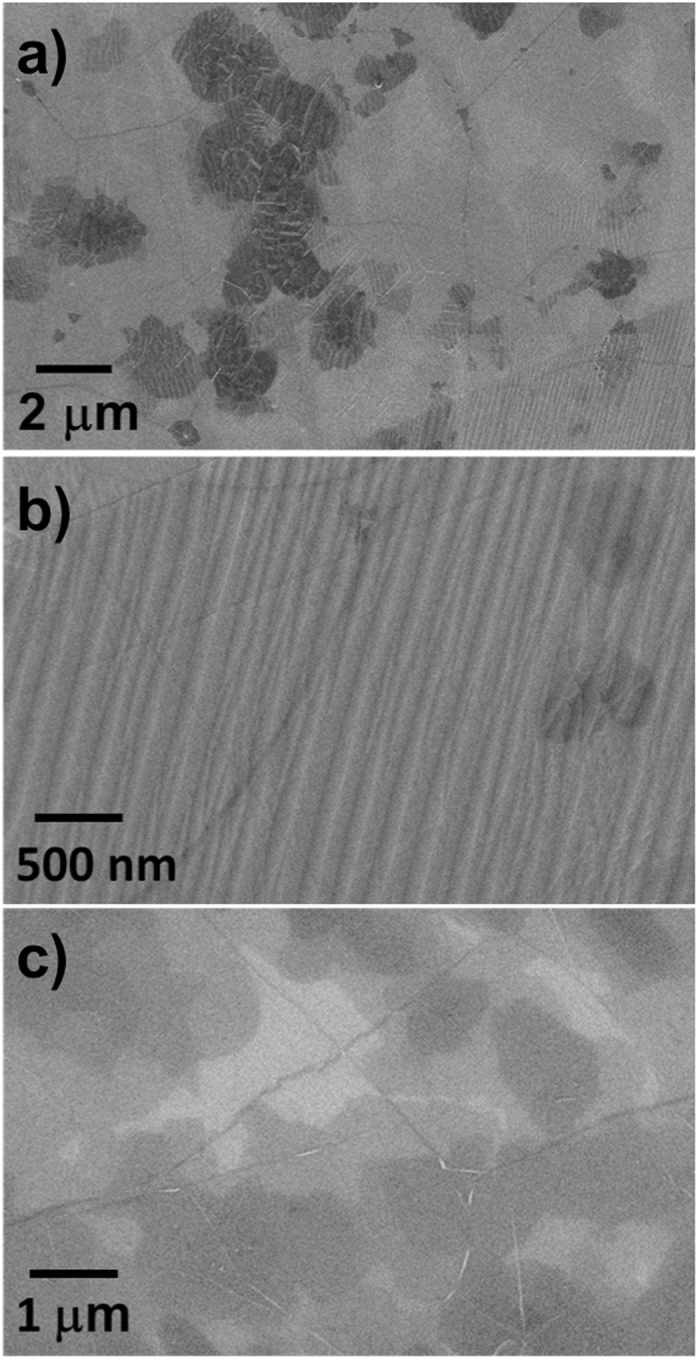
SEM micrographs at different magnification of graphene films grown in the optimized CVD reactor using the tantalum boat on Cu (**a**,**b**) and after transfer onto a Si/SiO_2_ substrate (**c**). No growth contaminants are visible at any magnifications.

We gained no direct evidence of a detectable contamination arising from the alumina boat, but it was subjected to cracking upon cooling after a few cycles. It has to be considered that the boat is not exposed to air while hot, unlike the furnace tube. Therefore, we deemed more appropriate to substitute it with a boat made of a refractory metal to further avoid possible contamination sources upon aging. As expected, the contaminants were observed to be fully suppressed by substituting the alumina boat with a tantalum (Ta) boat with a lid, built with an open “matchbox” geometry which allows the free circulation of the process gases over the copper foils, as schematized in Fig. 4. Ta is a refractory metal with an extremely high melting point of 2990 °C, which reaches a value of 3850 °C in case of its carburized form (TaC)^50^. Other than having high thermal stability, Ta is also not miscible with Cu (although miscible with Si) and can efficiently act as an oxygen getter, possibly further reducing SiO vapors, and can thus guarantee an unpolluted, stable CVD gaseous environment. Figure 5 shows SEM micrographs of graphene samples grown in our clean CVD system with the Ta boat. No white dots can be found anywhere contaminating the samples. A few secondary graphene domains are visible, but they are now randomly-oriented, proving that the nucleation is not driven by the presence of preferential sites, such as defects or contaminations.

**Figure 6:**
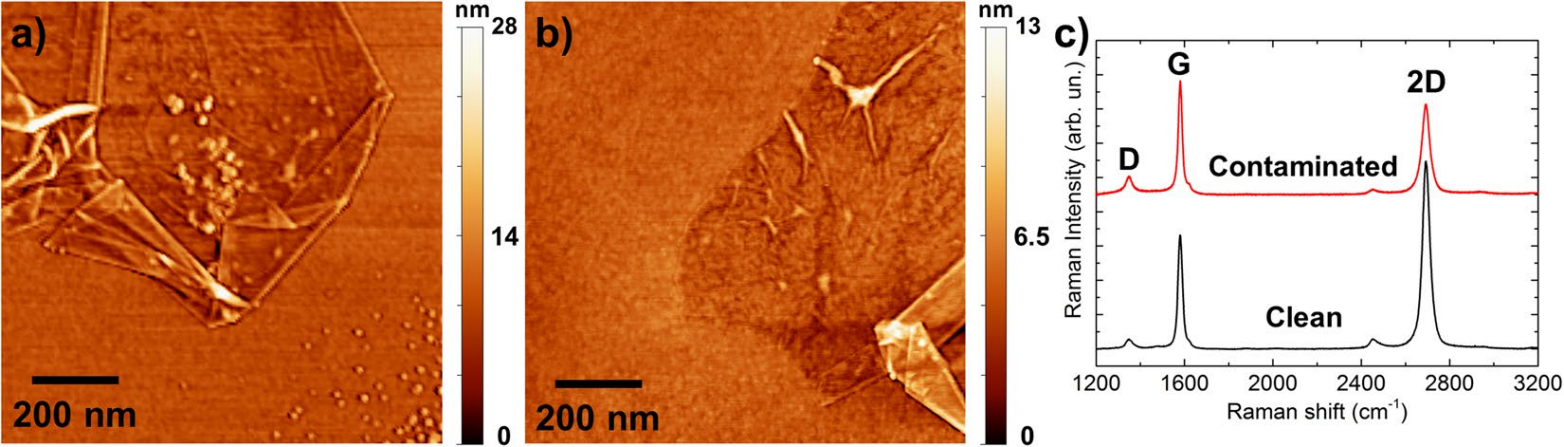
Representative AFM images of graphene transferred on Si/SiO_2_: (**a**) heavily contaminated sample grown in an aged quartz tube (>100 cycles), (**b**) clean sample grown in our modified CVD reactor. (**c**) Raman spectra of the two graphene samples (the D, G, 2D bands are peaked respectively at ~1348, 1580, 2692 cm^−1^).

The AFM images in Fig. 6 show graphene samples transferred on Si/SiO_2_ with a different degree of contamination depending on the CVD system used. The sample grown in our modified system is completely free of contamination. The comparison of the Raman spectra taken on the two samples (Fig. 6c) demonstrates the effect that the contamination has on graphene^52^. The I_D_/I_G_ ratios in case of the clean and contaminated samples are 0.08 and 0.16, respectively. The I_2D_/I_G_ ratios are 1.65 and 0.79, respectively. This proves that, as expected, the contamination worsens the graphene crystalline quality (and in turn its electron mobility) and promotes the undesired nucleation of secondary islands/layers^37, 53^, as also suggested by Figure S3.

**Figure 7:**
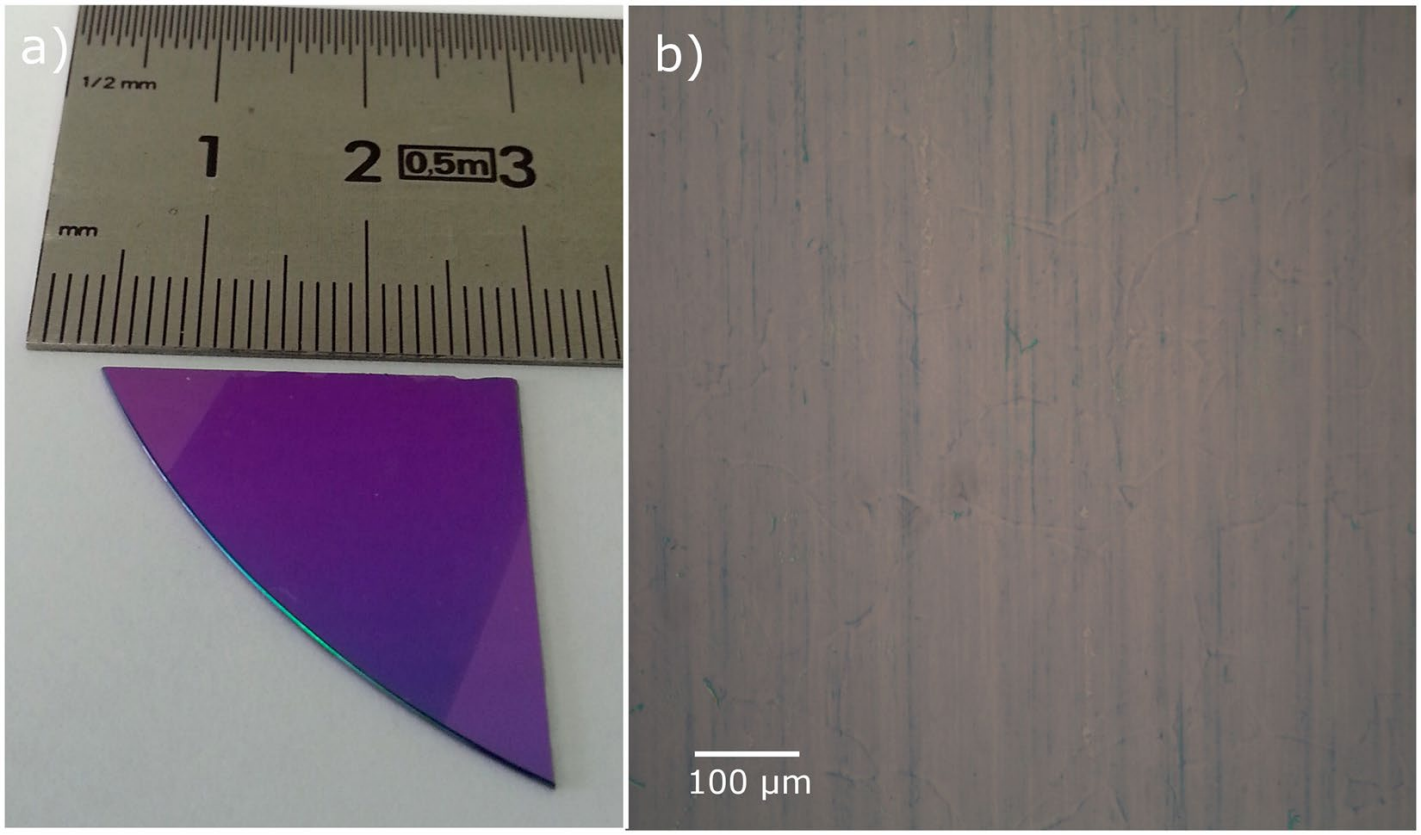
(**a**) Picture of graphene transferred onto Si/SiO_2_ (300 nm) and (**b**) optical micrograph of the sample.

By using our modified CVD system it is possible to grow and transfer clean graphene over cm-large areas, as shown in Fig. 7. The lack of oxide contaminants was observed in all the samples produced with this system after >100 growth processes^54^.

## Conclusion

Despite their widespread use, quartz tubes may not be the ideal choice for CVD furnaces dedicated to the growth of graphene on Cu substrates, since the presence of Cu vapors is expected to degrade the purity of the quartz tube. As Cu diffuses into the quartz, a phenomenon accompanied by its devitrification, SiO vapors are emitted from the tube walls and can severely contaminate the exposed growth surfaces. All these effects have a practical relevance in the CVD growth of graphene, since the ageing of quartz tubes and other components - such as sample holders - will introduce contamination and cause reproducibility issues in the graphene batches, with a large increase in the production cost if the quartz glassware must be often replaced. We here proposed a modified hot-wall CVD system based on a quartz tube furnace with an internal coaxial alumina screen tube and a tantalum sample holder. This CVD configuration successfully produce contamination-free graphene samples on Cu foils over extended period of time. The reactor and process presented in this work were used to perform more than 100 growth processes without the occurrence of heterogeneous sample pollution. Our novel system configuration coupled with fast graphene growth from ethanol CVD and cyclododecane assisted transfer provide a platform for the high-throughput production of pure graphene. This modified system would be also strategic for the CVD growth of large single-crystal graphene which is at present are actively sought after for electronic applications, since this process is usually performed in highly diluted carbon precursors over extended time (several hours) and it is possibly subjected to an extreme level of contamination.

## Methods

### Experimental

#### Graphene growth and transfer

**Figure 8:**
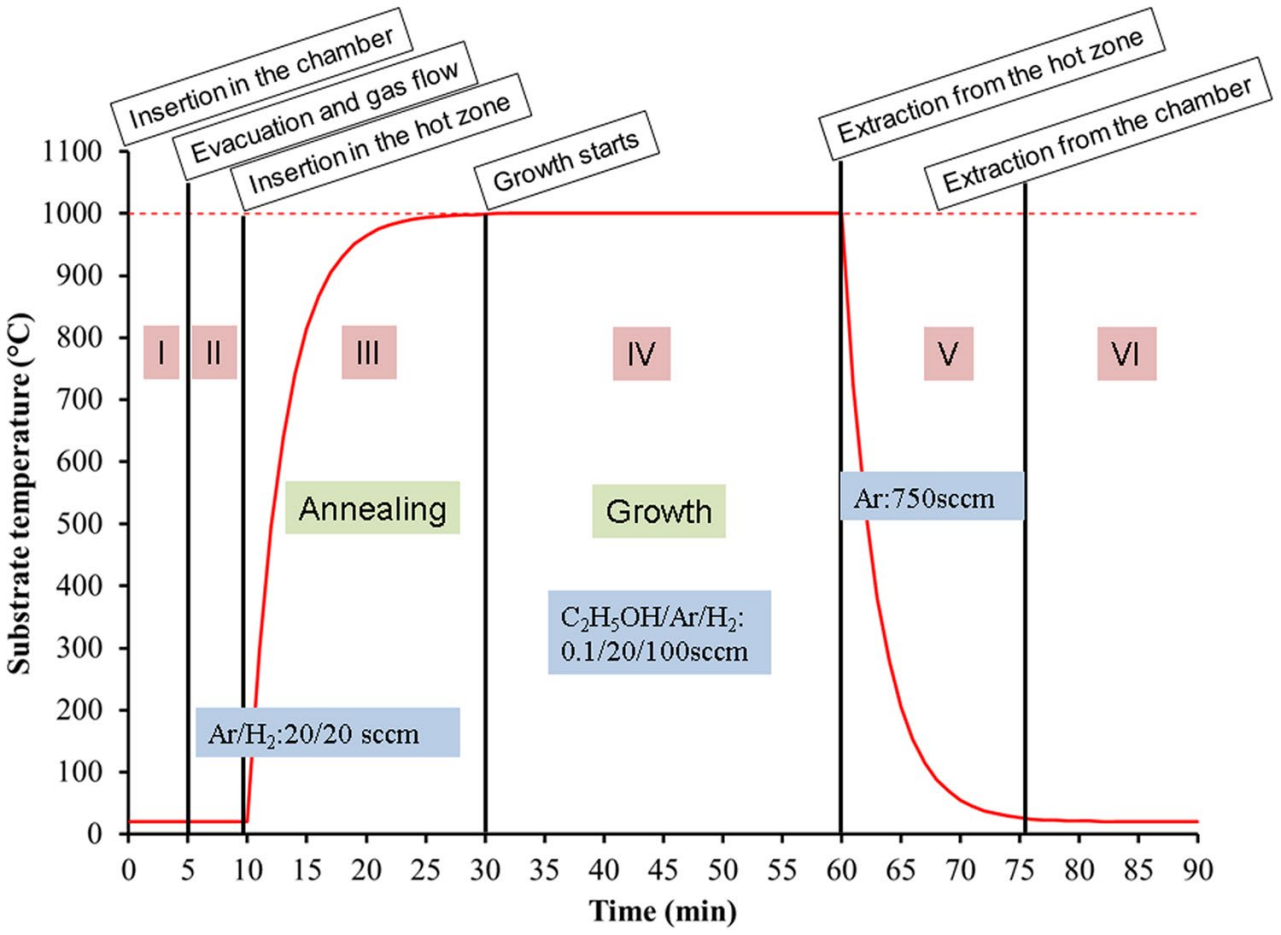
Temperature-time profile during a typical growth. (I) Insertion in the chamber, (II) evacuation and setting of the gas flows for annealing (Ar, H_2_), (III) insertion in the furnace hot zone and annealing, (IV) growth, (V) extraction from the hot zone and rapid cooling under Ar, (VI) filling with Ar and extraction from the chamber.

Graphene was synthesized on polycrystalline Cu foil (25 µm thick Cu-XLP/PHC, purity 99.95%) using low-pressure CVD. The Cu foil substrates were cut (2 × 2 cm^2^ substrates) and cleaned by ultrasonication in acetone and ethanol (15 min each). The details of the CVD system can be found in ref. 21. Briefly, it is based on a hot-wall quartz tube furnace which allows the rapid sample insertion/extraction in/from the hot zone without breaking the vacuum. A quartz boat acting as sample holder for the Cu substrates is inserted in the hot zone and the samples are annealed in Ar/H_2_ (20/20 sccm) for 20 min at 1000 °C. Afterwards, ethanol (C_2_H_5_OH) diluted in Ar (0.1% in 20 sccm of Ar) is introduced in the tube with 100 sccm of H_2_ for 30 min to carry out the graphene growth. Finally the sample holder is rapidly extracted from the hot zone and cooled down to room temperature under Ar flow. A typical graphene growth process is a six step process as summarized by the temperature-time diagram in Fig. 8. In this work we compare graphene grown in a system with all the components made of quartz (both wall reactor and sample holder) to graphene grown in a modified system designed to avoid the close exposure of the samples to quartz surfaces.

After the growth, graphene was transferred for analysis onto target substrates by the cyclododecane method^55, 56^. The complete removal of cyclododecane does not require solvents, but a mild thermal heating at 60 °C was used to speed up the sublimation.

#### Characterization methods

The presence of contaminants and the general surface morphology were investigated using a Zeiss (formerly LEO) 1530 field emission SEM, both on as grown samples and after their transfer. The analysis of samples transferred onto grids (Ni mesh with holey carbon film) was performed with a FEI Tecnai F20 ST TEM to determine the film microstructure. EDX was employed to determine the nature of the contaminants. By the examination of the transferred samples onto a Si/SiO_2_ wafer (with an oxide thickness of 300 nm) with an optical microscope^57^ the presence of holes and cracks due to the transfer process was evaluated. XRD patterns were recorded on a PANalytical Empyrean X-ray diffractometer equipped with a 1.8 kW Cu Kα ceramic X-ray tube, PIXcel^3D^ 2 × 2 area detector and operating at 45 kV and 40 mA. The diffraction patterns were collected in air at room temperature using Parallel-Beam (PB) geometry and symmetric reflection mode. High Temperature X-ray diffraction analysis (HTXRD) was performed using a Rigaku Smartlab system equipped with a 9 kW CuKα rotating anode (operating at 40 kV and 150 mA) and an Anton Paar DHS 900 domed hot stage, operating under vacuum conditions.

Raman measurements on the graphene sample (transferred on Si/SiO_2_ substrates) were collected with a Renishaw inVia confocal Raman microscope using an excitation line of 532 nm (2.33 eV) with a 50x objective lens, and an incident power of 1 mW on the samples (20 spectra collected and averaged for each sample).

## Electronic supplementary material

Supplementary Information
