# Supplementary material for: Contamination-free graphene by chemical vapor deposition in quartz furnaces
Source: Sci Rep. 2017 Aug 30;7:9927. doi: 10.1038/s41598-017-09811-z (PMC5577164; doi:10.1038/s41598-017-09811-z)
Supplement: Supplementary file 1 — Supplementary Information [file 41598_2017_9811_MOESM1_ESM.pdf]

Contamination-free graphene by chemical vapor deposition in quartz furnaces

Supplementary Information

Nicola Lisi[1,*], Theodoros Dikonimos[1], Francesco Buonocore[1], Martina Pittori[2,3], Raffaello Mazzaro[3,4], Rita Rizzoli[3], Sergio Marras[5], and Andrea Capasso[6,*]

[1]Energy Technologies Department (DTE), ENEA Casaccia, Via Anguillarese 301, 00123 Rome, Italy

[2]Department of Chemical Materials Environmental Engineering, Sapienza University of Rome, Via del Castro Laurenziano 7, 00161 Rome, Italy

[3]Institute for Microelectronics and Microsystems (IMM), National Research Council (CNR), Section of Bologna, Via Gobetti 101, 40129 Bologna, Italy

[4]Chemistry Department 'G. Ciamician', Bologna University, Via Selmi 2, 40126 Bologna, Italy

[5]Istituto Italiano di Tecnologia, Materials Characterization Facility, Via Morego 30, 16163 Genova, Italy

[6]Istituto Italiano di Tecnologia, Graphene Labs, Via Morego 30, 16163 Genova, Italy

*Corresponding authors: nicola.lisi@enea.it, andrea.capasso@iit.it

Graphene contamination

The contamination by white dots is not present on the graphene samples grown in a furnace equipped with new quartz tube and boat sample holder (**Figure S1**).

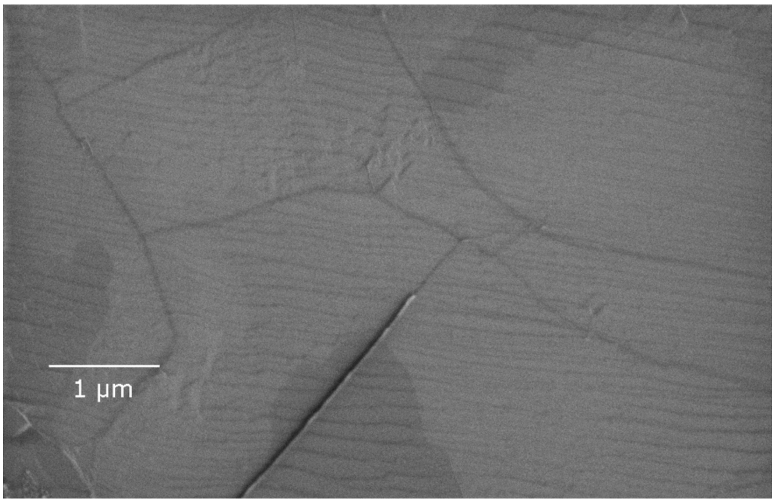

Figure S1. SEM image of uncontaminated graphene on copper using a CVD system with new quartzware.

High temperature XRD

In order to assess the occurrence of inversion in the copper contaminated quartz, a section of the tube (part B, **Figure 4**) was brought in vacuum to 900°C for 4 hours and then cooled down to room temperature, while its structure was monitored by in-situ XRD. The evolution of the XRD spectrum shows that both the α-cristobalite and α-quartz phases transform to the respective β-phase above 600°C and remain stable up to 900°C. This transformation is reversible and both β phases re-transform back to α below 600°C. As the “inversion” (α to β to α) of the tube during heating and cooling may favor copper diffusion in the bulk, we conclude that the irreversible devitrification (amorphous to cristobalite to quartz) is a slower process requiring much more than 6h.

It is not possible to ascertain, and it is pointless from the practical point of view, to which extent devitrification and the subsequent quartz crystalline phases promote the diffusion of copper inside the quartz bulk and enhance the release of SiO vapors.

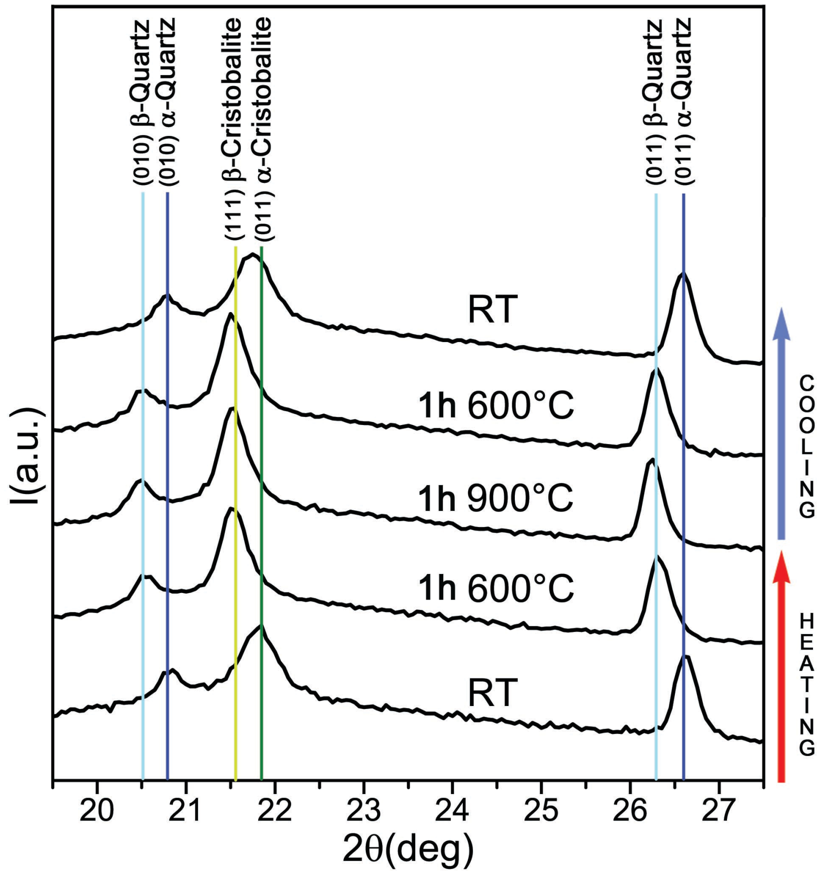

Figure S2. HTXRD patterns of the tube part B (860°C reached during the graphene growth process). The transition from α-quartz (trigonal, ICSD code: 156196) and α-cristobalite (tetragonal, ICSD code: 47221) to β-quartz (hexagonal, ICSD code: 89291) and β-cristobalite (cubic, ICSD code: 77460) respectively, observed during the heating cycle (starting from 600°C) is perfectly reversible. At the end of the cooling cycle, the integrated intensities of α-quartz and α-crystobalite peaks are exactly the same observed in the initial RT pattern.

Effects of the contamination on the growth process

In **Figure S3** a SEM micrograph of a contaminated copper surface is shown. It can be noticed that the SiO_x contaminations are often found in at the center of secondary nucleation graphene domains, indicating that aged quartz tube reactors may severely alter the growth of graphene.

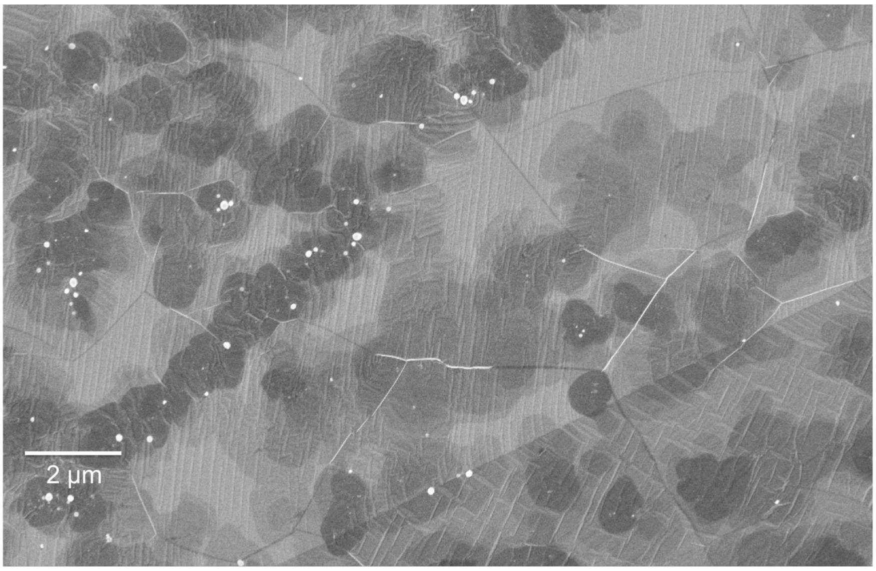

Figure S3 SEM micrograph showing graphene on copper grown in an aged quartz tube, highlighting the positiong of the SiO_x contaminants within secondary nucleation domains.

Effect of the sample holder and alumina CVD

When first placing the alumina tube screen, we found that also the quartz boat sample holder contributed to the contamination, since its use in conjunction with the alumina screen still yielded contaminated samples. In control experiments, the use of an old quartz tube with alumina boats also lead to contaminated growth, proving the all quartz glassware contributed to the contamination. Contrarily to the tube, the boat is sitting for most of the time in the hot zone and copper vapors do not condense as much on its surface as at the tube edges. Nevertheless, it is frequently extracted and inserted in the hot zone and copper was observed to diffuse in it (as a the pink taint), although not to the same extent as at the tube edges. Using

both the alumina screen and boat the contamination was significantly reduced but some small particles are still visible on the copper surface in **Figure S4**. We believe that this is because our alumina screen was not of the highest grade and it contained some SiO_2. It is nevertheless possible that a similar, although less severe, phenomenon of copper diffusion and interaction with alumina (Al_2O_3) leads to the formation of volatile compounds (AlO, Al_2O). When graphene is growth in a CVD system made of alumina tube and sample holder, the contamination is reduced with respect to quartz, due to the lower diffusion of Cu atoms into its lattice. Residual contaminants may be also due to the presence of some SiO_2 (<5%) in the alumina tube that was employed. the SEM images in this configuration are shown.

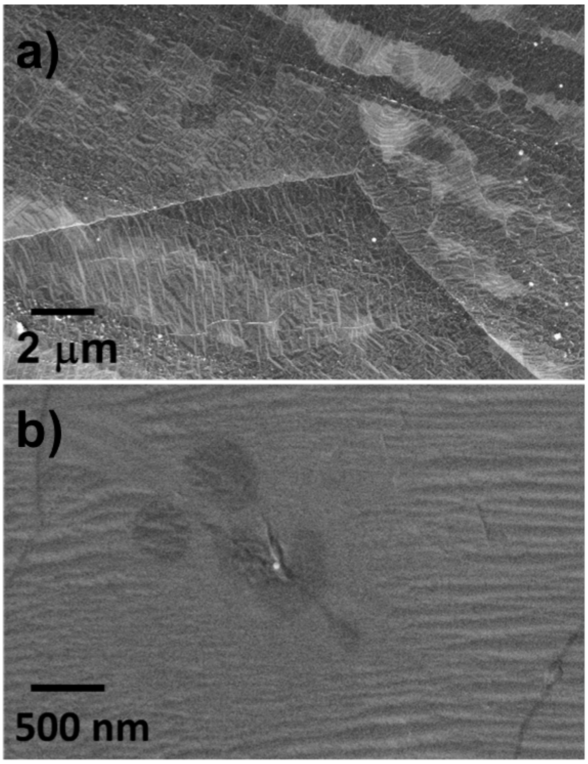

Figure S4. SEM images at different magnification of a graphene film grown in the CVD reactor using the alumina screen and boat. Contaminats are considerably reduced, but some white dots are still visible in a). Contamination-free areas can be found at higher magnification, as in b).
